# A New York Doctor

**Published:** 1882-11-20

**Authors:** 


A NEW YORK DOCTOR, worried, worn and weary
With dust and heat and circumstances dreary,
Resolved to rusticate and go a fishing;
And so he bids John tell all persons wishing
His counsel that a case of *Ichthyosis*
Has called him out of town in consultation,
His patients set him down a second Moses,
And propagate abroad their admiration.—*Pacific Med. Jour.*

A Georgia Doctor, worried, worn and often caught
By calls which ne'er a dime to his pocket brought,
Had in his house a room named *Billy Jones*,
Where, as a ruse, he oft did rest his weary bones;
And when his servant Jack answered to a call
From one of doubtful means, or none at all—
Especially if in his bed the Doctor snug and warm
Heard the loud hello! out in the dismal storm—
Quoth Jack in meek if not regretful tones—
"The Doctor will spend the night at *Billy Jones'*."

SOUTHERN MEDICAL RECORD.

